# Apixaban and oral implications

**DOI:** 10.4317/jced.52470

**Published:** 2015-10-01

**Authors:** Monica Lopez-Galindo, Jose V. Bagán

**Affiliations:** 1 Associate Professor, PhD, Dentistry Department, European University of Valencia, Valencia, Spain; 2 Dentistry Department, University of Valencia, Valencia, Spain. Chairman of Oral Medicine, University of Valencia. Head of the Department of Stomatology and Maxillofacial Surgery,Valencia University General Hospital, Valencia, Spain

## Abstract

**Background:**

Thrombotic disorders remain a leading cause of death in the Western world, and in this regard a number of anticoagulation treatment have been used, including heparins, fondaparinux, vitamin K antagonists (warfarin, acenocoumarol), and new oral anticoagulants such as apixaban. For years there has been great controversy regarding the use of anticoagulants in planning dental treatments that imply bleeding. The main concerns about using new oral anticoagulants in invasive dental procedures are bleeding due to the lack of an antidote, and the thrombotic risk of the disease for which anticoagulation was indicated in the first place.

**Material and Methods:**

A literature search was conducted through May 2014 using the keyword “apixaban” for publications in the ISI Web of Knowledge. The search was extended to other databases (PubMed, Scopus and the Cochrane Library).

**Results:**

Based on the results of the different studies, apixaban seems to be a good alternative to conventional anticoagulation and a reasonable treatment option, though its main and most common adverse effect is bleeding. Dose adjustment is needed in some patients, though regular laboratory monitoring is not required. The use of the drug in different patient populations will define its final indications and doses.

**Conclusions:**

Regarding the use of apixaban in the dental setting, there is a compelling need for further clinical studies in order to establish more evidence-based guidelines for patients requiring antithrombotic treatment.

** Key words:**Apixaban, dental treatment, dental implications.

## Introduction

Thrombotic disorders remain a leading cause of death in the Western world, and in this regard a number of anticoagulation treatment have been used, including unfractionated heparin, low molecular weight heparins (semuloparin, enoxaparin), fondaparinux, vitamin K antagonists (warfarin, acenocoumarol) and new oral anticoagulants (1).

Heparins in general and fondaparinux require subcutaneous administration. Vitamin K antagonists can be administered via the oral route but have other drawbacks such as drug and diet interactions, a slow onset of therapeutic effects, the need for regular International Normalized Ratio (INR) monitoring and dose adjustments, and a narrow therapeutic index (2).

The new oral anticoagulants, including apixaban, do not have these disadvantages (3).

Apixaban is an orally active, direct selective factor Xa inhibitor. It reversibly and selectively inhibits the activation site of factor Xa (4,5), a trypsin-like serine protease which is the final enzyme in the coagulation cascade, being responsible for fibrin clot formation. Factor Xa serves as the link between the extrinsic and intrinsic coagulation pathways. It exerts antithrombotic and anti-coagulant effects by diminishing the conversion of prothrombin to thrombin (6). Apixaban indirectly inhibits platelet aggregation by reducing thrombin generation (7). It has a favorable safety and efficacy profile also in special populations (elderly subjects, obese individuals, patients with renal and liver dysfunction) (8), and can be administered with or without food (4).

Direct inhibition of factor Xa attenuates the generation of thrombin, whilst direct inhibition of thrombin affects its activity. Accordingly, the inhibition of factor Xa, produced for example by apixaban, preserves hemostatic function (9).

Apixaban has high oral bioavailability (approximately 52%), and reaches peak plasma concentrations approximately three hours after administration, with a half-life of about 12 hours. It is predominantly eliminated through metabolic pathways and non-renal mechanisms, with a minimal potential for drug interactions and the formation of reactive metabolites (4,9). About 25% of the drug is excreted in urine (10). The pharmacokinetic profile of apixaban is compatible with dosing twice or once a day (11).

Cytochrome P450 isoenzyme 3A4 (CYP3A4) and sulfotransferase F1A1 appear to be the major enzymes involved in metabolizing apixaban to an inactive circulating metabolite. Strong inhibitors of CYP3A4 (such as macrolide antibiotics, protease inhibitors and azole antifungals) substantially increase the drug levels and should not be taken in combination with apixaban (6). However, the effect of apixaban is independent of vitamin K intake (12).

There seems to be little evidence on the reversal of the effects of apixaban (13).

For years there has been great controversy regarding the use of anticoagulants in planning dental treatments that imply bleeding. The main concerns about using new oral anticoagulants in invasive dental procedures are bleeding due to the lack of an antidote, and the thrombotic risk of the disease for which anticoagulation was indicated in the first place (14).

Apixaban may represent a good alternative to traditional anticoagulation, though due consideration is required not only of the benefits but also of the possible inconveniences associated with its use.

## Objetive

To evaluate the use of apixaban in the dental setting before and after dental extraction and/or surgery.

## Material and Methods

A literature search was conducted through May 2014 using the keyword “apixaban” for publications in the ISI Web of Knowledge. No lower limit was set. The search resulted in 403 papers, and was then limited to articles in English and preferably clinical studies in adult patients (≥ 18 years of age). The search focused on clinical trials, but other articles were also taken into account for relevant information about apixaban.

The search was extended to other databases (PubMed, Scopus and the Cochrane Library). The PubMed search involved “apixa-ban” in the title, and the activated filters were: clinical trial, humans, and English in the advanced search. The Scopus search in turn involved “apixaban” in the title, abstract and keywords in the advanced search, with restriction to the English language. Lastly, the Cochrane Library search was activated with the word “apixaban” from 2009 to 2014.

Regarding the relationship between apixaban and dentistry, other searches were performed in the ISI Web of Knowledge, PubMed, Scopus and the Cochrane Library, using the words “apixaban” and “dental treatment”, “apixaban” and “oral consequences”, and “apixaban” and “dental implications”.

The main issue in dental practice is to design a protocol for using the drug, trying to establish whether apixaban should be suspended or not when performing dental extraction, followed by reintroduction after the surgical procedure.

## Results

Apixaban has been used for preventing thromboembolic events in acutely ill non-surgical patients (15) and following knee or hip replacement surgery (16,17), as well as in patients with acute coronary syndrome (ACS) (18) and in cases of atrial fibrillation (AF) (19,20).

Why is it so important to prevent thromboembolic events? Venous thromboembolism (VTE) is a common complication in surgical patients and also in acutely ill non-surgical patients. It is also a potentially fatal complication (21). Patients undergoing orthopedic surgery (hip or knee replacement) are at a high risk of developing postoperative VTE complications. The provision of thromboprophylaxis after hospital discharge has reduced in the rates of symptomatic and asymptomatic VTE (22). In this regard, correct anticoagulation is essential in order to decrease patient morbidity and mortality (23).

Apixaban has been used on a prophylactic basis in patients undergoing hip or knee replacement surgery to reduce the incidence of VTE. Despite such preventive measures, however, symptomatic VTE occurs in 2-4% of the cases during the first three months after surgery, and subclinical deep-vein thrombosis is recorded in approximately 15-20% of patients soon after surgery (23).

Several clinical trials on apixaban have been published since 2007, starting with the APPROPOS study, in which the drug was used to prevent VTE 12-24 hours after total knee replacement surgery. A total of 1238 patients participated in this randomized, multicenter, 8-arm, parallel group phase II trial (11) ( Table 1).

**Table 1:**
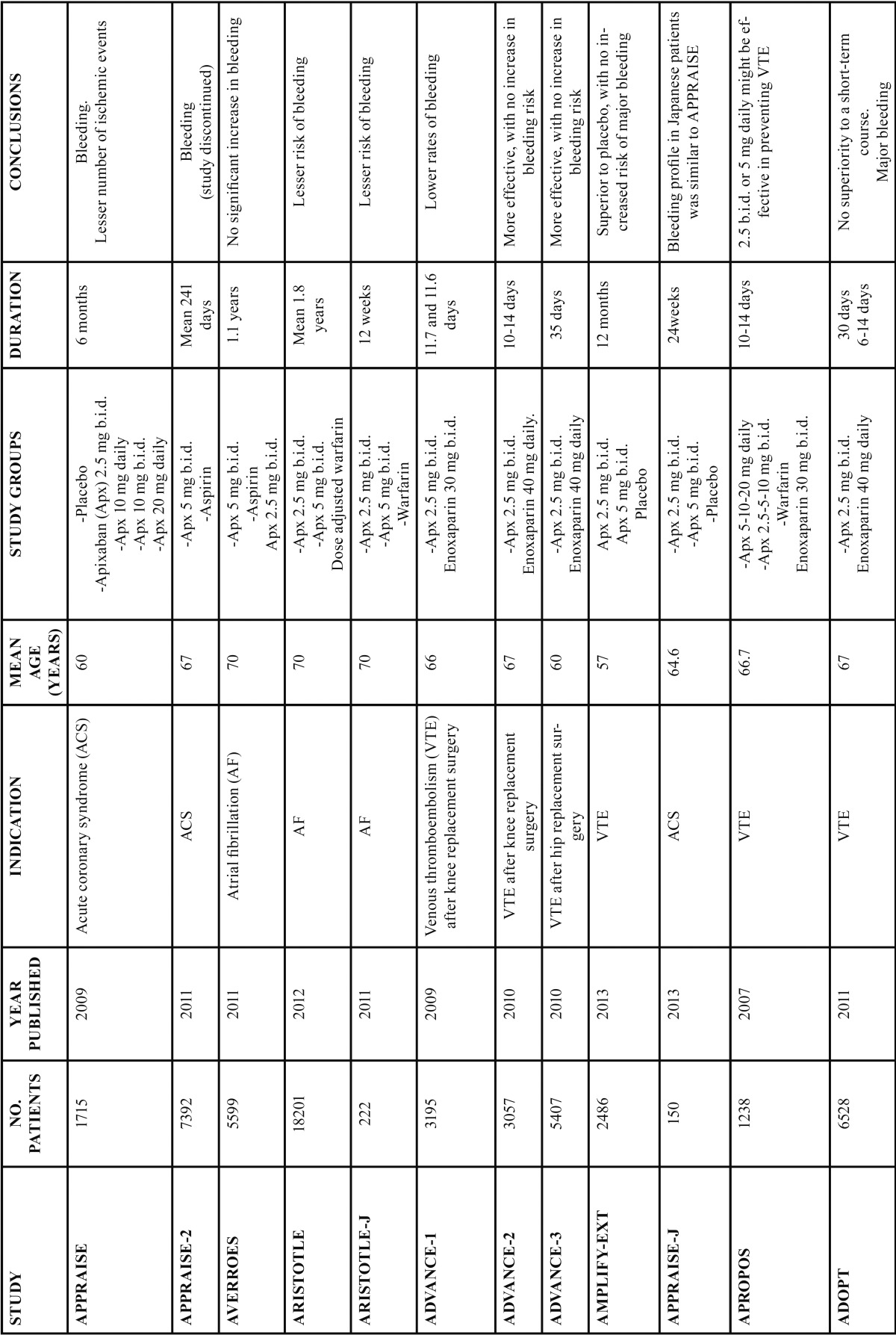
Studies with Aproxiban.

Other studies on the prevention of thromboembolic complications after VTE have been the ADVANCE (2009) , ADVANCE-2 (2010) and ADVANCE-3 (2010) studies, and the ADOPT trial (2011), involving the largest number of patients to date (n=6528). The AMPLIFY-EXT trial in turn was published in 2012, with the participation of 2486 patients. The ADVANCE, ADVANCE-2 and ADVANCE-3 trials included 3195, 3057 and 5407 patients, respectively (15-17,24,25).

Most of the studies on the prevention of thromboembolic complications after VTE have been phase III trials (ADVANCE, AD-VANCE-2 and ADVANCE-3). The exception has been the APPOPOS study, which was a phase II trial. AMPLIFY-EXT and ADOPT failed to specify the trial phase involved (11,15-17,24,25).

The mean patient age in the different trials was 66 years (ADVANCE), 67 years (ADVANCE-2), 60 years (ADVANCE-3), 67 years (ADOPT) and 57 years (AMPLIFY-EXT). In the APPROPOS trial the mean patient age was 66.7 years.

In most of the mentioned studies, apixaban was used to prevent thromboembolism after knee (ADVANCE, ADVANCE-2, AP-PROPOS) or hip replacement surgery (ADVANCE-3), or in acutely ill non-surgical patients (ADOPT). In the AMPLIFY-EXT study, apixaban was used in patients with VTE who had completed 6-12 months of anticoagulation therapy and for whom there was a clinically balanced decision between either continuing or interrupting anticoagulation therapy.

Regarding the treatment used in each trial, the patients received oral apixaban at a dose of 2.5 mg twice a day (b.i.d.) or enoxaparin via the subcutaneous route at doses of 30 mg b.i.d. (ADVANCE) or 40 mg once a day (ADVANCE-2, ADVANCE-3, ADOPT). Several apixaban doses were used in the 8-arm APROPOS study (5, 10 and 20 mg once a day, as well as 2.5, 5 and 10 mg b.i.d.). Warfarin was also administered (INR 1.8-3) to another group of patients. Enoxaparin was used subcutaneously at a dose of 30 mg b.i.d. The most recent study, the AMPLIFY-EXT trial, only involved two different apixaban doses of 2.5 or 5 mg b.i.d., without comparison versus enoxaparin.

The duration of treatment was 10-14 days in the ADVANCE, ADVANCE-2 and APPROPOS trials, and was a little longer in the ADVANCE-3 study (35 days). The ADOPT trial contemplated treatment for 30 days in the apixaban group and for 6-14 days in the enoxaparin group. The longest treatment duration corresponded to the AMPLIFY-EXT study (12 months).

Regarding the results obtained by the different studies in the prevention of VTE, the authors of the APROPOS trial concluded that apixaban might be effective in preventing VTE after total knee replacement surgery, administered at doses of 2.5 mg b.i.d. or 5 mg daily. This phase II trial led to selection of the apixaban dose used in the phase III ADVANCE trial.

The conclusion of the ADVANCE study on the prevention of VTE after knee replacement surgery was that apixaban resulted in fewer cases of clinically relevant bleeding than enoxaparin, with a similar adverse events profile.

In the ADVANCE 2 trial, apixaban 2.5 mg b.i.d. was seen to be more effective than enoxaparin 40 mg daily, with no increase in bleeding risk (or with a similar incidence of major bleeding), in preventing VTE after knee replacement surgery.

In the ADVANCE 3 study, the results were similar to those obtained in the ADVANCE 2 trial - apixaban being associated to lower VTE rates, with no increase in bleeding.

In the AMPLIFY-EXT study, the findings suggested apixaban to be superior to placebo in reducing VTE, with no increased risk of major bleeding.

In the ADOPT trial (involving non-surgical patients), an extended course of apixaban to prevent thrombosis was not found to be superior to a shorter period with enoxaparin. Apixaban was associated with significantly more major bleeding events than enoxaparin. However, this trial did not provide evidence justifying extended prophylaxis in a broad population of non-surgical patients after hospital discharge.

Regarding the use of apixaban in acute coronary syndrome (ACS), three studies should be commented: the APPRAISE, APPRAISE 2 and APPRAISE-J trials. Acute coronary syndrome includes both acute myocardial infarction and unstable angina, and is a worldwide leading cause of cardiovascular mortality (26).

Patients with ACS continue to experience recurrent ischemic events despite revascularization and ongoing antiplatelet therapy. Other management alternatives, including apixaban, are therefore investigated (27).

The APPRAISE trial was published in 2009 and involved 1715 patients with a mean age of 60 years. This was an international, multicenter, double-blind, randomized, placebo-controlled, dose-ranging study and the largest global phase II trial published up until then. It was designed to assess the efficacy and safety of apixaban in preventing recurrent ischemic events after ACS (27).

The APPRAISE 2 trial in turn was published in 2011, and a total of 7392 patients took part. The mean age was 67 years. This was a double-blind, randomized, placebo-controlled, phase III trial comparing apixaban with placebo, in addition to antiplatelet therapy, in patients with recent ACS and at least two additional risk factors for recurrent ischemic events (28).

The APPRAISE-J study was published in 2013, and comprised a limited sample of 150 Japanese patients. This was a placebo-controlled, randomized, double-blind phase II study conducted at the same time as the APPRAISE 2 trial and involving a design similar to that of the APPRAISE study (26).

The APPRAISE study was the first phase II trial to explore several apixaban doses in patients with ACS administered concomitant treatment in the form of aspirin in most cases (≤ 165 mg per day) and clopidogrel. The clopidogrel dose was not specified. The apixaban doses were 2.5 mg b.i.d., 10 mg once daily, 10 mg b.i.d., or 20 mg daily (27).

In the APPRAISE 2 trial, apixaban was used at a dose of 5 mg b.i.d. compared with placebo and added to standard antiplatelet therapy (aspirin or aspirin plus any P2Y12 receptor antagonist - predominantly clopidogrel). Some patients with an estimated creatinine clearance of < 40 ml/min. at the time of randomization were randomly assigned to receive 2.5 mg of apixaban b.i.d. or matching placebo (28).

In the APPRAISE-J trial, the patients received apixaban 2.5 or 5 mg b.i.d. or matching placebo, as well as standard antiplatelet therapy (aspirin or aspirin plus a thienopyridine such us clopidogrel or ticlopidine) (26).

The duration of the treatment differed in each study. Therapy in the APPRAISE trial lasted 26 weeks (a little over 6 months), versus 241 days (8 months) in the APPRAISE 2 study and 24 weeks (a little under 6 months) in the APPRAISE-J trial.

A dose-related increase in bleeding was observed in the APPRAISE study, together with a trend towards fewer ischemic events following the addition of apixaban to antiplatelet therapy in patients with recent ACS. In the APPRAISE 2 trial, 5 mg of apixaban b.i.d., added to antiplatelet therapy in high risk patients after ACS, resulted in an increased number of major bleeding events, without a significant reduction in recurrent ischemic phenomena. This study was terminated prematurely as a result. In the APPRAISE-J trial, the bleeding profile with apixaban was similar to that found in the APPRAISE study; it was terminated before completion not because of the results of the study itself, but because of the recommendations of the DSMC (Data and Safety Monitoring Committee) for the concurrent APPRAISE-2 trial.

The prevention of thromboembolic events in patients with atrial fibrillation (AF) is crucial, because these individuals are at an increased risk of stroke (29,30). Atrial fibrillation is the most common chronic arrhythmia (6). Patients with AF have a higher risk of developing heart failure, poorer quality of life, and cognitive impairment (6), i.e., AF and its potential complications are associated to increased mortality and morbidity risk (12,31), and constitute a significant health and economic problem (32). Chronic kidney disease carries a high risk of cardiovascular disease, including AF (33). Some authors have concluded that the oral new anticoagulants are more cost-effective than warfarin, and apixaban has been pointed to as the preferred drug in this respect (34). Other authors have reported the new oral anticoagulants to be more efficacious than warfarin in preventing stroke and systemic embolism in patients with AF (35,36).

The studies addressing AF have been the AVERROES and ARISTOTLE-J trials published in the year 2011. The ARISTOTLE study (2012) was the trial with the largest number of patients (n=18,201. The ARISTOTLE-J study was a phase II and partially blinded trial, whereas ARISTOTLE and AVERROES were double-blind, double-dummy phase III trials. The three studies were randomized, and the mean patient age was 70 years (23-25). The duration of the trials was 12 weeks in the case of the ARISTOTLE-J study, 1.1 years for AVERROES, and 1.8 years in the case of the ARISTOTLE trial.

The treatment administered in the three mentioned studies was apixaban 2.5 mg b.i.d. or 5 mg b.i.d.. Apixaban was compared with aspirin (18-324 g per day) in the AVERROES trial, dose-adjusted warfarin (to achieve target INR 2-3) in the ARISTOTLE study, and dose-adjusted warfarin in the ARISTOTLE-J trial (to achieve target INR 2-3, or 2.0-2.6 in the case of age ≥ 70 years).

The results obtained in the AVERROES trial showed apixaban to be superior to aspirin, with a reduction of the risk of stroke or systemic embolism, and with no significant increase in the risk of major bleeding or intracranial hemorrhage. The clear benefit of apixaban led the DSMC to recommend early termination of the study. In the ARISTOTLE trial, apixaban was found to be superior to warfarin, causing less bleeding and resulting in lesser mortality. In the ARISTOTLE-J trial, apixaban was seen to be well tolerated, with fewer cases of major and clinically relevant non-major bleeding versus warfarin over 12 weeks.

We have found very few results on new oral anticoagulants in relation to dental practice. There seems to be a genuine need for clinical studies, with a view to establishing management protocols for these drugs. The risk posed by new oral anticoagulants in dentistry is associated to procedures in which bleeding is involved, such as dental extractions, dental and/or periodontal surgery, etc. As general rules in the dental setting, it is preferable for surgery to be done in the morning rather than in the afternoon, and preferably at the start of the week, in order to offer the patient adequate management in the case of bleeding. Dental extraction should be as scantly traumatic as possible. After extraction, hemostatic measures must be adopted to help bleeding control, such as absorbable hemostatic dressings in the form of oxidized cellulose (Surgicel®), collagen sponges (Haemocollagel®), or reabsorbable gelatin sponges (Spongostan®). It is also advisable to suture the extraction socket and apply compressive gauzes impregnated with tranexamic acid (14,37).

The main inconvenience with the use of apixaban is the lack of an antidote (13,38).

## Discussion

Regarding the use of apixaban for preventing thromboembolic complications after VTE, ACS or AF, the most widely accepted dosage appears to be 2.5 or 5 mg b.i.d. The only uncertainty in this regard is deciding which is the most convenient dose.

The trials on the prevention of thromboembolic complications after VTE had a duration of 10-14 days, 35 days or 12 months, i.e., covering a broad range. The trials in which apixaban was used in application to ACS had a treatment duration of about 6 months, while the administration of apixaban in AF lasted approximately 12 months (20 months in the AVERROES trial). The duration of the treatments varies greatly among the different trials, and this may influence assessment of the usefulness of apixaban in a positive or negative way. In this regard, doubts may arise as to whether such durations of treatment are sufficient or, contrarily, if long-term use of the drug may give rise to clinical side effects.

In some cases, standard antiplatelet therapy (aspirin or aspirin plus thienopyridine) is added to apixaban, as in the APPRAISE, APPRAISE 2 or APPRASE-J trials, and it would be very useful to know whether such therapy is necessary or not. Based on the trials using apixaban for the prevention of ACS, it seems that major bleeding events occurred without a significant reduction in recurrent ischemic events; this is why some trials were interrupted before completion. It appears that antiplatelet therapy should not be added in these cases.

With respect to the studies on use of apixaban in AF, the drug was compared with aspirin or warfarin, and was not used concomitantly with these agents. The results referred to apixaban were encouraging and reflected clear benefit of apixaban over aspirin or warfarin or enoxaparin.

When new oral anticoagulants are administered to patients requiring dental procedures that imply bleeding, several aspects should be taken into account: the invasiveness of the dental procedure, the half-life of the drug, renal function (39), comorbidities and the risk of thromboembolic events (37). The invasiveness of the dental treatment is related to bleeding risk; according to Spyropoulos (40), minor bleeding risk is found in simple extractions (< 3 teeth) and surgery lasting less than 45 minutes, while major bleeding risk is found in multiple extractions (> 3 teeth), surgery lasting more than 45 minutes, and head and neck cancer surgery. In relation to renal function, van Ryn *et al.* (39) used creatinine clearance (ml/min) to define direct anticoagulant suspension times. They established that for a half-life of 13 hours (apixaban has a half-life of about 12 hours), the time from last drug dose to surgery is 24 hours for lesser bleeding risk and 2-4 days for high bleeding risk. According to a first approach made by Spyropoulos and Douketis (40), the reintroduction of apixaban in the case of low bleeding risk surgery should be 24 hours post-surgery, at a dose of 5 mg b.i.d. In the case of high bleeding risk surgery, apixaban should be resumed 2-3 days after surgery at a dose of 5 mg b.i.d., with the possibility of reducing the dose to 2.5 mg b.i.d. in patients at high risk of thromboembolism.

## Conclusions

Based on the results of the different studies, apixaban seems to be a good alternative to conventional anticoagulation and a reasonable treatment option, though its main and most common adverse effect is bleeding. Dose adjustment is needed in some patients, though regular laboratory monitoring is not required. The use of the drug in different patient populations will define its final indications and doses.

Regarding the use of apixaban in the dental setting, there is a compelling need for further clinical studies in order to establish more evidence-based guidelines for patients requiring antithrombotic treatment.
